# Comparison of ketorolac intravenous versus acetaminophen intravenous in treating headache following head trauma: a semi-experimental study

**DOI:** 10.1186/s41016-024-00381-4

**Published:** 2024-10-21

**Authors:** Behzad Zohrevandi, Marjan Hosseinnia, Niloufar Balikshahi, Masoud Jobaneh, Ehsan Kazemnezhad Leili, Naema Khodadadi-Hassankiadeh

**Affiliations:** 1 Guilan Road Trauma Research Center, Trauma Institute, Guilan University of Medical Sciences, Rasht, Iran; 2 Department of Clinical and Administrative Sciences, School of Pharmacy, Notre Dame of Maryland University, Baltimore, MD USA; 3 Guilan University of Medical Sciences, Rasht, Iran; 4 Department of Health in Disasters and Emergencies, School of Public Health and Safety, Shahid Beheshti University of Medical Sciences, Tehran, Iran; 5 Guilan University of Medical Sciences, Rasht, Iran

**Keywords:** Acetaminophen, Trauma, Headache, Ketorolac

## Abstract

**Background:**

Post-traumatic headache is a disabling secondary headache disorder often attributed to traumatic brain injury and affects millions of individuals worldwide. Few studies have been done on the treatment needs of these patients in emergency departments. The purpose was to compare the effectiveness of ketorolac intravenous versus acetaminophen intravenous in reducing headaches in patients following head trauma.

**Methods:**

This was a semi-experimental study in which the participants were assigned two groups. In the acetaminophen intravenous group, 1 g acetaminophen and in the ketorolac intravenous group, 60 mg of this drug was injected. Statistical analysis was done with IBM SPSS statistical software version 21, and a P-value less than 0.05 was considered statistically significant.

**Results:**

Among samples after 6 h from the injection, the pain score in the ketorolac intravenous group was less than the acetaminophen intravenous group (*P* = 0.006). Also, the pain reduction rate in the ketorolac intravenous group was more than the acetaminophen intravenous group from before the injection until 2 h after it (*P* = 0.01) and before injection until 6 h after it (*P* = 0.001). The frequency of drowsiness in 2 and 6 h after drug administration in the ketorolac intravenous group was lower than the acetaminophen intravenous group, which is significant in 2 h after drug administration (*P* = 0.038). The verbal analog scale score comparison for two groups 2 h before medicine administration with pain control score (*P* = 0.03) and 6 h with pethidine use control (*P* = 0.003) is significant.

**Conclusions:**

According to this study, ketorolac’s intravenous effect on pain control is better than that of acetaminophen intravenous. With more samples, we can express the survey results more decisively in the future.

## Background

In more than 50 to 70% of cases, the leading cause of death due to trauma is brain damage [1, 2]. Posttraumatic headache is a disabling secondary headache disorder often attributed to traumatic brain injury and affects millions of individuals worldwide [3, 4]. Population-based data have shown that the lifetime prevalence of this disorder is estimated to be 4.7% in men and 2.4% in women [4, 5]. Headache is the most common complaint in emergency departments [4, 6].

This headache is a heterogeneous disorder, and patients may respond to different and specific treatments. The lack of evidence-based approaches has forced clinicians to choose treatment methods for primary headaches (migraines and tension headaches). A concerted effort to address these shortcomings is to conduct randomized controlled trial studies. This approach, in turn, leads to a better description of the disease and the availability of evidence-based treatment options [4].

In developing countries, morphine is mainly used in emergencies for pain relief in trauma patients despite known side effects. At the same time, acetaminophen and ketorolac intravenous (IV) are more suitable painkillers for trauma patients. However, the preference for the last two drugs is not clear. They consider that both drugs have their strengths and weaknesses [7]. Ketorolac is a nonsteroidal anti-inflammatory drug (NSAID) that is used to reduce pain, fever, and inflammation; its use can be a substitute for the use of narcotic painkillers, and respiratory depression does not follow these drugs [8–10].

Recently, the FDA approved acetaminophen IV as an NSAID antipyretic and analgesic drug. This drug is faster and more effective than oral or rectal [10, 11]. Studies have shown that acetaminophen IV has fewer side effects than opioids are preferred. Complications such as nausea, emesis, pruritus, hypotension, respiratory depression, constipation, and hepatotoxicity are less after the administration of this drug [12, 13]. The results of a systematic review showed that acetaminophen IV has better pain reduction than IV morphine sulfate, especially in acute limb trauma and traumatic headache. However, more studies need to be done so that emergency medicine specialists can make better decisions about headache management in patients with head trauma [13].

A few studies have been conducted to compare the comparative efficacy of prescribing ketorolac IV vs. acetaminophen IV for headaches following head trauma. In this study, we aimed to test the application of ketorolac IV vs. acetaminophen IV for managing patients with trauma injuries to support clinicians in better understanding these technologies and choosing the better treatment choice.

## Methods

### Study design

The comparison study was conducted at Porsina Hospital in Rasht after receiving the code of ethics from the Ethics Committee of Guilan University of Medical Sciences. In this study, 60 patients who were admitted to the emergency department of the hospital with complaints of headache following head trauma after clearly explaining the objective, risks, and benefits of the study and obtaining a written consent form participation were randomly assigned into 2 groups of 30 people: ketorolac IV and acetaminophen IV. The Health Ethics Research Guilan University of Medical Sciences (I.R.) reviewed this study protocol GUMS.REC.1394.13.

### Patient population

Persian language speaker patients aged more than 18 years old with a documented history of headaches following head trauma were admitted to the emergency department of the hospital. They had a level of consciousness of 15. No abnormal findings were seen in medical examinations, and imaging was included.

Brain internal and systemic diseases, which mainly include metabolic lesions such as kidney, liver, gland, and connective tissue diseases, vasculitis, infectious diseases, treatment with antidepressants, sleeping and psychoactive drugs, and drug or alcohol abuse, were not included in the study. Exclusion criteria were drug allergy, decreased level of consciousness, and patient withdrawal at any time.

### Randomization

The random method of selecting patients was that the letter A indicated acetaminophen IV and the letter K indicated ketorolac IV which was poured into a bag in equal numbers. Then, the person under study took one of the papers; in this order, the patient’s injection drug was chosen. One g of acetaminophen IV was injected into group A, and 60 mg of ketorolac IV was injected intravenously into group B. The patients were evaluated before starting the drug and at 2, 6, and 12 h after receiving the drug. If they needed an extra dose of the drug to relieve pain, the same dose of pethidine was injected equally into both groups. Also, two groups were matched in terms of the distribution of sociodemographic variables.

### Protocol

In the case of a patient admitted to the emergency room with a headache following head trauma, a trained medical student from the research team took the history and reviewed the patient’s records to check the patient’s eligibility. For patients whose neurologic examinations and imaging (MR images and CT scans), electroencephalography, laboratory tests, and lumbar puncture or consultation with various medical specialists confirmed that they were not brain injured, informed consent is obtained before any data collection and after random allocation, and they are determined to belong to which group. Patients received either 1 g of acetaminophen IV diluted in 100 ml of normal saline or 60 mg of ketorolac IV via slow IV infusion. All the drugs used were selected from the same manufacturer. UNI-PHARMA S.A. manufactured Apotel, and Exir Pharmaceutical Co. manufactured ketorolac. The pain score was determined based on the verbal analog (VAS) pain scale (10 reported the worst imaginable pain, and 0 was pain-free) pre- and post-intervention. The same medical information checked the VAS score, and side effects were recorded in a predetermined questionnaire based on age, gender, occupation, mechanism of trauma, and side effects (pruritus: yes/no, drowsiness: yes/no, nausea/emesis based on scoring from 1 to 4 (1 = no nausea and emesis, 2 = nausea without emesis, 3 = emesis less than twice, 4 = severe emesis more than twice), need for pethidine consumption, and measured time (2-h pre- and 2-, 6-, and 12-h postdrug prescription).

### Statistical analysis

Quantitative data were displayed as mean, standard deviation, and qualitative data as frequency. The chi-squared test and Fisher’s exact test were used to compare qualitative data, and an independent *T*-test was used to compare quantitative variables between two groups if they followed a normal distribution. If they did not follow a normal distribution, Mann–Whitney’s nonparametric equivalent tests were used. Repeated measure ANOVA was used to investigate the trend of changes in VAS score, and ANCOVA was used to control the effects of pethidine consumption due to the studied drugs on VAS. Analyses were done with SPSS version 21. A significant level was considered with *P* < 0.05.

## Results

Sixty eligible patients were allocated to 2 groups of 30 and entered the study. There were 18 men and 12 women in the ketorolac IV group and 16 men and 14 women in the acetaminophen IV group. The average age in the ketorolac IV group was 29.8 ± 9 and in the acetaminophen IV group was 30.4 ± 10.27. Also, the occupation of most people in the ketorolac IV group was university student and in the acetaminophen IV group was a housewife. There were no significant differences between the two groups in terms of sociodemographic characteristics, including gender, age, job, and mechanism of trauma (*P* > 0.05) (Table 1).

**Table 1:** Demographic data of patients in ketorolac IV vs. acetaminophen IV groups

Parameter		Ketorolac IV(*n*=30)	Acetaminophen IV (*n*=30)	*p*-value
Male		18 (60)	16 (53.3)	0.662
Age		29.8 ± 9	30.4 ± 10.27	0.777
Job	Employment	7 (23.3)	3 (10)	0.690
	Farmer	1 (3.3)	2 (6.7)	
	Worker	0 (0)	1 (3.3)	
	Business	6 (20)	5 (16.7)	
	Housewife	5 (16.7)	9 (30)	
	Student	9 (30)	7 (23.3)	
	Unemployed	1 (3.3)	2 (6.7)	
	Soldiers	1 (3.3)	1 (3.3)	
Trauma mechanism	Car -car	12 (40)	16 (53.3)	0.54
	Car-motorcycle	1 (3.3)	0 (0)	
	Motorcycle	6 (20)	3 (10)	
	Pedestrian	1 (3.3)	0 (0)	
	Violence	4 (13.3)	6 (20)	
	Falling	6 (20)	5 (16.7)	

Values are mean ± SD or number of patients

The two groups were the same regarding the baseline VAS before the treatment, and there was no statistically significant difference (*P* = 0.787). Significant differences were based on VAS in the ketorolac IV vs. acetaminophen IV group 6 h after injection (*P* = 0.006). Moreover, the trend of VAS score reduction was significant in preinjection times (baseline) to 2 h (*P* = 0.01) and baseline to 6 h (*P* = 0.001) after injection in the ketorolac IV group vs. the acetaminophen IV group. It should be noted that the comparison of VAS scores and other outcomes could not be made due to the drop of 100% and 90% 12 h after injection in the two groups of ketorolac IV and acetaminophen IV (Table 2).

**Table 2:** Comparison of mean VAS of pain decreased based on VAS in the ketorolac vs. IV acetaminophen group

Pain	Group	*N*	Mean	SE	*p*-value
VAS baseline	Ketorolac	30	6.77	1.43	0.787
	Acetaminophen	30	6.67	1.42	
VAS 2 h	Ketorolac	30	2.07	1.78	0.108
	Acetaminophen	30	2.9	2.16	
VAS 6 h	Ketorolac	29	0.48	0.74	0.006
	Acetaminophen	29	1.45	1.66	
VAS baseline to 2 h	Ketorolac	30	4.7	1.29	0.01
	Acetaminophen	30	3.77	1.43	
VAS baseline to 6 h	Ketorolac	29	6.34	1.08	0.001
	Acetaminophen	29	5.17	1.39	
VAS 2 6 h	Ketorolac	29	1.66	1.2	0.589
	Acetaminophen	29	1.48	1.21	

*VAS* Visual analog scale

### Comparing side effects

The frequency of pruritus 2 h after injection was the same in the ketorolac IV group vs. the acetaminophen IV group and was not statistically significant (*P* = 0.313). At other times, pruritus was 0% in both groups.

The frequency of drowsiness 2 and 6 h after injection was lower in the ketorolac IV group vs. the acetaminophen IV group, but it was statistically significant only 2 h after the infusion (*P* = 0.038). At other times, drowsiness in both groups was the same and insignificant.

The frequency of nausea and emesis 2 and 6 h after injection was the same in the ketorolac IV group vs. acetaminophen IV group and was not statistically significant (*P* < 0.05). In 12 h after the injection, the occurrence of nausea and emesis was 0% in both groups.

The frequency of pethidine consumption 2 h after injection was the same in the ketorolac IV group vs. the acetaminophen IV group and was not statistically significant (*P* = 0.692). At 6 and 12 h after injection, pethidine consumption was 0% in both groups (Table 3).

**Table 3:** Comparison of the frequency of side effects in the two study groups according to measurement times

Variable		Group				
		Acetaminophen IV		Ketorolac IV		*p*-value
		*N*	%	*N*	%	
Pruritus in 2 h	Yes	0	0	1	3.3	0.313
	No	30	100	29	26.7	
	Total	30	100	30	100	
Pruritus in 6 h	Yes	0	0	0	0	-
	No	29	100	29	100	
	Total	29	100	29	100	
Pruritus in 12 h	Yes	0	0	0	0	-
	No	5	100	3	100	
	Total	5	100	3	100	
Drowsiness in 2 h	Yes	4	13.3	0	0	0.038
	No	26	86.7	30	100	
	Total	30	100	30	100	
Drowsiness in 6 h	Yes	2	6.9	0	0	0.15
	No	27	93.1	29	100	
	Total	29	100	29	100	
Drowsiness in 12 h	Yes	0	0	0	0	-
	No	5	100	3	100	
	Total	5	100	3	100	
Nausea and emesis in 2 h	Yes	5	16.7	4	13.3	0.718
	No	25	83.3	26	86.7	
	Total	30	100	30	100	
Nausea and emesis in 6 h	Yes	4	13.8	2	6.9	0.389
	No	25	86.2	27	93.1	
	Total	29	100	29	100	
Nausea and emesis in 12 h	Yes	0	0	0	0	0
	No	5	100	3	100	
	Total	5	100	0	100	
Pethidine consumption in 2 h	Yes	6	20	5	16.7	0.692
	No	24	80	25	83.3	
	Total	30	100	30	100	
Pethidine consumption in 6 h	Yes	0	0	0	0	-
	No	29	100	29	100	
	Total	29	100	29	100	
Pethidine consumption in 12 h	Yes	0	0	0	0	-
	No	5	100	3	100	
	Total	5	100	3	100	

Although both in the ketorolac IV group (*P* < 0.0001) and in the acetaminophen IV group (*P* < 0.0001) based on the repeated measure ANOVA statistical method, a downward trend can be seen, and the changes in the pain score between all measurement times are significant in both groups based on the Bonferroni test (*P* < 0.0001), and the type of decreasing trend in the two groups was not the same so that the slope of the reduction of pain score in the ketorolac IV group is more than the acetaminophen IV group (*P* < 0.0001) (Table 4).

**Table 4:** ANOVA analysis of comparison of changes trend in pain score between the measurement times in two groups

Group	Time 1	Time 2	VAS	SE	P^b^
Ketorolac IV	Baseline	2 h	4.69	0.244	0.0001
		6 h	6.345	0.2	0.0001
	2 h post-injection	6 h	1.655	0.223	0.0001
Acetaminophen IV	Baseline	2 h	3.690	0.258	0.0001
		6 h	5.172	0.258	0.0001
	2 h post-injection	6 h	1.483	0.225	0.0001

^b^Bonferroni test

The results of ANCOVA analysis comparing the VAS score of two groups in 2 h with the adjusted pain score pre-injection (*P* = 0.039) (Table 5) and comparing the VAS score of the two groups in 6 h with the adjusted pethidine consumption in 2 h (*P* = 0.003) were significant (Table 6).

**Table 5:** ANCOVA analysis of comparing the VAS of two groups in 2 h by adjusting the pain score pre-injection

Sources of effect	SS	df	σ^2^	F	*p*-value
Modified model	66.448	3	22.149	7.273	0.0001
Constant	374.668	1	374.668	123.032	0.0001
Group	13.63	1	13.63	4.476	0.039
Pain score before injection	50.03	1	50.03	16.429	0.0001
Interaction of group-pain score before injection	6.001	1	6.001	1.971	0.166
Error	170.536	56	3.045		
Total	607	60			
Total modified	236.983	59

**Table 6:** ANCOVA analysis of comparing the VAS of two groups in 2 h by adjusted pethidine consumption

Sources of effect	SS	df	*σ*^2^	F	*p*-value
Modified model	46.339	3	15.446	13.997	0.0001
Constant	84.395	1	84.395	76.476	0.0001
Group	10.31	1	10.31	9.343	0.003
Pain score pre-injection	31.263	1	31.263	28.33	0.0001
Interaction of group-pain score pre-injection	0.732	1	0.732	0.663	0.419
Error	59.592	54	1.104		
Total	160	58			
Total modified	105.931	57			

### Summary of results

Based on this study’s data, ketorolac’s effect on pain control is better than acetaminophen. However, the results of this study were in the form of a pilot, and the power of the study test in Comparison of pain in 2 hours is equal to 54.7%, and 6 hours after drug administration is equal to 85.1%. With more samples, the research results can be expressed more strongly in the future.

## Discussion

Effective management of headaches following head trauma by emergency physicians is critical. Using effective drugs with fewer complications improves clinical results and safety for patients complaining of headaches following head trauma [14].

The results of the present study showed that the pain score 6 h after injection in the ketorolac IV group was lower than in the acetaminophen IV group, and the pain reduction rate from baseline to 2 h and 6 h after drug administration in the ketorolac IV group was more than the acetaminophen IV group. A similar study showed significant declines in VAS scores in ketorolac IV and paracetamol throughout the time sequence (*P* < 0.05). The statistical VAS score was slightly higher in the paracetamol group at most time points, except for 6 h [15]. According to a previous study, ketorolac IV and acetaminophen IV produced a similar postoperative palliative effect. However, ketorolac reduced Dilaudid usage and improved the return of bowel function compared to acetaminophen [16]. In another study, there was no significant difference between the two groups of ketorolac IV vs. acetaminophen IV in the average scores of VAS after surgery. However, ketorolac IV produced lower pain scores than acetaminophen IV in the later postoperative treatment [10]. The results of Sorri et al.’s (2021) study showed that there is no significant difference in pain reduction between the three groups treated with acetaminophen and ketorolac IV and a combination of the two drugs [14].

In examining the changes in the pain score during the study, although in the ketorolac IV vs. acetaminophen IV group, a downward trend can be seen among all measurement times; however, the type of decreasing trend in the two groups was not the same. Hence, the slope of the pain score reduction in ketorolac IV is more than in the acetaminophen IV group. Sorri et al. (2021) showed that the hill of pain reduction in the combination of ketorolac IV and acetaminophen IV was more than in either alone. It is suggested that if there are no contraindications, the combination of acetaminophen IV and ketorolac IV should be injected to reduce the pain of conscious trauma patients [14].

The frequency of pruritus, nausea and emesis, and pethidine consumption in all the hours under examination after drug administration was insignificant in the ketorolac IV vs. acetaminophen IV group. The frequency of drowsiness 2 h after drug administration was lower in the ketorolac IV vs. acetaminophen IV group. In Anand et al.’s (2013) study, the incidence of nausea in the ketorolac IV group was significantly lower compared to the acetaminophen IV [10]. In the study comparing IV ibuprofen vs. ketorolac IV for reducing renal colic pain, the most common complication was nausea and emesis, which was more in the IV ibuprofen group. However, there was no significant difference between the two drugs regarding side effects [17]. Zackova et al. (2001) also showed that the ketorolac IV group had lower side effects, such as emesis [18]. There was no significant difference in pain reduction between ketorolac and dexamethasone. However, in the dexamethasone group, the percentage of patients who needed narcotics and antiemetics was significantly lower [19]. Side effects caused by the use of narcotic drugs, such as respiratory depression, nausea, and drowsiness, do not occur with the use of ketorolac, so ketorolac is reported to be a safe and effective drug for pain control in patients after surgery and other pain cases [20]. Some studies report other side effects of ketorolac, including coagulation, gastrointestinal problems, and nephrotoxicity [21], which were not investigated in the present study. However, studies have shown that the use of ketorolac can reduce opioid injection use and its consequences [22, 23]; moreover, the use of injectable ketorolac for pain relief of joints, its effectiveness, is good. It has few short-term side effects [24]. In the study of Javaherforooshzadeh et al. (2020), there were significant differences in morphine consumption in the two paracetamol versus ketorolac IV groups at 24 h and 48 after intubation, and the average consumption of morphine was higher in the ketorolac IV group [15].

In the present study, the comparison of the VAS score of the two groups in 2 h with the adjusted pain score pre-injection and the comparison of the VAS score of the two groups in 6 h with the adjusted pethidine consumption in 2 h were significant. Based on this study’s data, ketorolac’s effect on pain control was better than the acetaminophen IV group. In Rahimzadeh et al.’s (2013) study, the number of additional painkillers used to control pain to the satisfaction of the patient was lower in the acetaminophen IV group than in the ketamine group [25].

However, in the present study, ketorolac IV vs. acetaminophen IV had better effectiveness with fewer side effects. Previous studies have reported that ketorolac IV is as effective as morphine or meperidine in relieving postoperative pain. Due to ketorolac’s good effectiveness in pain, the need to receive pethidine similar to acetaminophen IV has not decreased.

### Limitations

With more samples, the study results can be expressed more strongly in the future. For the discussion section, there was a lack of studies that compared the efficacy and side effects of these two drugs in headaches following head trauma. So, there is a need for further research in this field, and it is suggested that the following studies, especially in terms of investigating the side effects caused by the drug, should be done prospectively.

Further research is needed to assess whether a combined approach of IV acetaminophen and IV ketorolac may negate the need for opioid use.

## Conclusion

The present study showed that ketorolac IV was more effective than acetaminophen IV in reducing headaches following head trauma. The results of the current study emphasize controlling the pain associated with head trauma with non-narcotics with more efficacy and fewer side effects at a reasonable cost.

## Data Availability

The data used and analyzed during this study can be obtained from the corresponding author upon reasonable request.

## Abbreviations

NSAID: Nonsteroidal anti-inflammatory drug
IV: Intravenous
vs: Versus
VAS: Verbal analog pain scale
